# Effect of Surface Treatment on Enamel Cracks After Orthodontic Bracket Debonding: Er,Cr:YSGG Laser-Etching Versus Acid-Etching

**Published:** 2017-09

**Authors:** Hassanali Ghaffari, Amirhossein Mirhashemi, Tahereh Baherimoghadam, Amir Azmi, Reza Rasooli

**Affiliations:** 1 Assistant Professor, Department of Orthodontics, School of Dentistry, Shahed University of Medical Sciences, Tehran, Iran; 2 Associate Professor, Dental Research Center, Dentistry Research Institute, Tehran University of Medical Sciences, Tehran, Iran; Department of Orthodontics, School of Dentistry, Tehran University of Medical Sciences, Tehran, Iran; 3 Assistant Professor, Department of Orthodontics, School of Dentistry, Yasuj University of Medical Sciences, Yasuj, Iran; 4 Dentist, Private Practice, Shiraz, Iran

**Keywords:** Acid Etching, Dental, Dental Enamel, Lasers, Orthodontic Brackets

## Abstract

**Objectives:**

This study sought to compare enamel cracks after orthodontic bracket debonding in the surfaces prepared with erbium, chromium: yttrium-scandium-galliumgarnet (Er,Cr:YSGG) laser and the conventional acid-etching technique.

**Materials and Methods:**

This in-vitro experimental study was conducted on 60 sound human premolars extracted for orthodontic purposes. The teeth were randomly divided into two groups (n=30). The teeth in group A were etched with 37% phosphoric acid gel, while the teeth in group B were subjected to Er,Cr:YSGG laser irradiation (gold handpiece, MZ8 tip, 50Hz, 4.5W, 60μs, 80% water and 60% air). Orthodontic brackets were bonded to the enamel surfaces and were then debonded in both groups. The samples were inspected under a stereomicroscope at ×38 magnification to assess the number and length of enamel cracks before bonding and after debonding. Independent-samples t-test was used to compare the frequency of enamel cracks in the two groups. Levene’s test was applied to assess the equality of variances.

**Results:**

No significant difference was noted in the frequency or length of enamel cracks between the two groups after debonding (P>0.05).

**Conclusions:**

Despite the same results of the frequency and length of enamel cracks in the two groups and by considering the side effects of acid-etching (demineralization and formation of white spot lesions), Er,Cr:YSGG laser may be used as an alternative to acid-etching for enamel surface preparation prior to bracket bonding.

## INTRODUCTION

Brackets are used in fixed orthodontics to force the teeth to move in three dimensions. The introduction of direct bracket bonding revolutionized orthodontic treatments; however, establishing a sufficiently strong bond to enamel to keep the brackets in place during the entire course of treatment, yet not too strong to damage the enamel upon debonding, has remained a challenge [1]. The bond between the bracket and enamel is based on mechanical interlocking of the adhesive into the micro-porosities of the enamel surface.

Therefore, a successful bond requires precise enamel surface preparation [2]. The introduction of the acid-etching technique enabled bonding of orthodontic brackets to the enamel surface. Some modifications have been made in this technique to accelerate the procedure and decrease the extent of enamel demineralization [3]. At present, enamel preparation with acid-etching is the gold standard in orthodontic treatments and 30% to 50% phosphoric acid gel, applied for 30 to 60 seconds, is commonly used for this purpose. Although removing the interprismatic mineral structure of the enamel surface by acid-etching and creating a rough surface enhances the retention of adhesive resins, the treated enamel becomes more susceptible to caries. Acid-etching removes the superficial protective enamel layer, making the teeth more vulnerable to long-term acid attacks. This problem is magnified when the acid-etched surface is not entirely covered by resin or is exposed to saliva before resin application [4]. Thus, researchers have long been in search of alternative conditioning methods to overcome the disadvantages of acid-etching with a phosphoric acid etchant. Surface treatment with erbium, chromium: yttrium-scandium-gallium-garnet (Er,Cr:YSGG) laser has been suggested as an alternative method to achieve this purpose. Although Er,Cr:YSGG laser was introduced to dentistry for ablation of hard and soft dental tissues, its sub-ablative irradiation has been proposed as an alternative to acid-etching of enamel and dentin. It seems that laser-etching is a suitable alternative to acid-etching of enamel since it is painless and creates no vibration or heat. Additionally, laser-etching of enamel creates micro-porosities that are perfect for resin penetration [5]. Due to the benefits of laser-etching over the acid-etching technique, the former is becoming increasingly popular for routine clinical use [6]. Carbon dioxide (CO_2_) laser irradiation alters the calcium-phosphate ratio and confers resistance to the enamel against acid attacks [7]. Moreover, laser-etching is time-saving since water-spraying and air-drying are not required in Er,Cr:YSGG laser-etching; therefore, the risk of salivary contamination during rinsing and drying is eliminated [8]. In 2007, Basaran et al [9] evaluated the shear bond strength, enamel surface characteristics, and mode of failure of orthodontic brackets bonded to enamel after acid-etching and laser-etching with Er,Cr:YSGG laser and reported that the mean shear bond strength and enamel surface texture obtained by laser-etching were comparable to those attained by acid-etching. In 2008, Ozer et al [6] evaluated the shear bond strength of orthodontic brackets and the surface properties and adhesive remnant index (ARI) of the enamel surfaces prepared with Er,Cr:YSGG laser and acid-etching with a phosphoric acid etchant, and found no significant difference between the two methods. In 2011, Basaran and colleagues [10] studied the shear bond strength between orthodontic brackets and enamel following Er,Cr:YSGG laser irradiation and reported that the efficacy of Er,Cr:YSGG laser was comparable to that of the acid-etching technique. Enamel cracks are distinct and fissure-like lines in the enamel surface that cannot be detected clinically in most cases [11]. Orthodontic treatment can cause enamel cracks [12]. In 2005, Zachrisson and Buyukyilmaz [13] reported that vertical enamel cracks and, with a slightly lower frequency, oblique and horizontal cracks were present in half of the orthodontically treated teeth in young adults. An increase in the number of enamel cracks after orthodontic treatment may be due to a natural increase unrelated to orthodontic bracket debonding or to the force exerted during treatment or upon debonding [14]. Generally, bracket debonding after orthodontic treatment can cause enamel cracks [13]. Considering the relatively recent introduction of Er,Cr:YSGG laser as an alternative to acid-etching for orthodontic bracket bonding, studies on its effect on the enamel surface and the frequency of cracks after bracket debonding are scarce and the available experiments have mainly focused on the bond strength between brackets and enamel following Er,Cr:YSGG laser irradiation [6,9,10], and not on the frequency of cracks after debonding. Considering the gap of information in this respect, this study sought to assess and compare enamel cracks after orthodontic bracket debonding in the surfaces prepared with Er,Cr:YSGG laser-etching and conventional acid-etching techniques.

## MATERIALS AND METHODS

This in-vitro experimental study was conducted on 60 human premolars, freshly extracted due to orthodontic purposes. The sample size was calculated using two-sample t-test power analysis procedure of PASS 11 software program (SPSS Inc., Chicago, IL, USA).

The inclusion criteria consisted of maxillary and mandibular premolars of patients aged 13–19 years with a normal anatomical form and sound enamel, without any cracks, fractures, caries or fluorosis, and with no history of surface treatment with chemical agents (such as bleaching treatment with hydrogen peroxide). The specimens were evaluated under a stereomicroscope (SNZ1000, Nikon, Tokyo, Japan) at ×38 magnification to ensure that all the teeth met the inclusion criteria. The teeth were stored in saline at 4°C for one month.

### Microscopic examination of the enamel surface before bracket bonding:

**Fig. 1:**
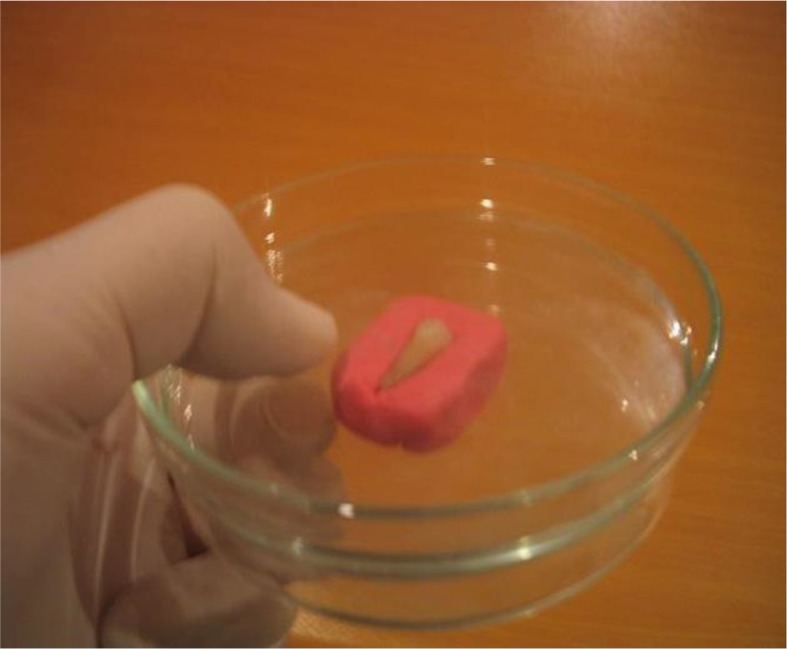
Applying pressure on the tooth by a second plate in order to position the buccal surface parallel to the horizon

To standardize the viewing conditions under the microscope, each tooth was mounted in a modeling dough on a plate while another plate of the same size was compressed over it in order to position the buccal surface parallel to the horizon (Fig. 1). The cracks and their directions were observed under the stereomicroscope at ×38 magnification with light illumination.

As recommended by Pickett et al [15], the teeth were rotated 360° around the central point of their buccal surfaces; otherwise, the cracks in the same direction as the light rays could not be visualized. The length of the cracks on the surfaces of 10 samples was measured by a ruler on the images transferred to a computer.

**Fig. 2:**
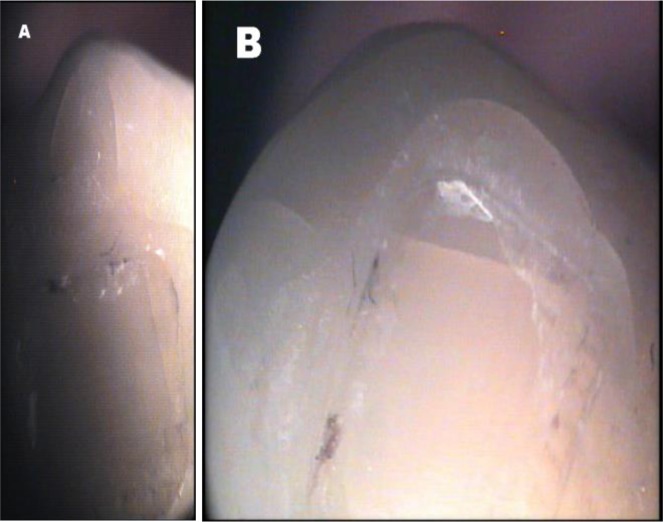
Enamel cracks and their location, direction, and length. (a) Before bracket bonding. (b) After debonding

The cracks that were not in the form of a straight line were divided into smaller straight lines with different directions. The lengths of these small segments were measured and added to obtain the entire crack length. By considering the magnification parameters and the distance between the lens and the tooth surface, the length of each unit of the ruler was calculated to be 62.5μm. Thus, the length of the cracks was initially calculated in microns and was then converted to millimeters. After evaluating the structural pattern of the buccal surface of each tooth, the number and length of enamel cracks were recorded by two observers from the Anatomy Department and the Histomorphometry and Stereology Research Center of Shiraz University of Medical Sciences. Each crack was allocated a number (Fig. 2a).

In order to standardize the conditions, the number and length of cracks after debonding were recorded by using the previously described method and by the same observers (Fig. 2b). The microscope was connected to a computer equipped with a digital camera (Sony, Tokyo, Japan).

The two observers analyzed the images by the Stereolith (version 2) software program (Shiraz University of Medical Sciences, Shiraz, Iran). The length of each crack was measured using the point-sampled intercepts method [16]. All the samples were evaluated by the two observers and the mean Of the values reported by them was calculated (the interobserver intraclass correlation coefficient or ICC=0.92). Since there was no difference between the lengths of the cracks in the 10 samples measured both by a ruler and by the Stereolith software program (ICC=1), measurements in the remaining samples were made only by using the Stereolith software program.

### Bracket bonding:

The teeth were randomly divided into two groups of 30 for laser-etching (group A) and acid-etching (group B). In group A, the teeth were etched with 37% phosphoric acid gel (Gel ETCH, 3M Unitek, Monrovia, CA, USA) for 15 seconds, were rinsed for 15 seconds, and were air dried for 15 seconds. In group B, the teeth were irradiated with Er,Cr:YSGG laser (Waterlase iPlus, Biolase Inc., Irvine, CA, USA) for 60 microseconds by a gold handpiece with MZ8 tip (0.8 mm in diameter) operated at the 2780nm wavelength, 50Hz frequency and 4.5W output power with 80% water and 60% air.

After surface preparation in both groups, a thin layer of primer (Transbond XT, 3M Unitek, Monrovia, CA, USA) was applied on the enamel in both groups and was cured for 20 seconds using a light-curing unit (PenCure LED light, Morita, Kyoto, Japan). After applying the adhesive resin (Transbond XT light cure adhesive paste; 3M Unitek, Monrovia, CA, USA) on the bracket base (standard edgewise brackets with a 0.018-inch slot; DynaLock, 3M Unitek, Monrovia, CA, USA), the bracket was placed at the center of the buccal surface of each tooth [10, 17]. An explorer was used to place the bracket on the enamel surface by a uniform force and also to remove the excess adhesive. Light-curing was done for 20 seconds. The teeth were stored in distilled water for 48 hours to prevent dehydration prior to debonding.

### Bracket debonding:

The brackets were debonded using bracket-removing pliers (Dentaurum, Ispringen, Germany), according to the manufacturer’s instructions. A shear peeling force was applied by the pliers to the bracket wings until they were detached from the enamel surface.

### Microscopic examination of the enamel surface after bracket debonding:

By using the digital camera connected to the stereomicroscope and the Stereolith software program, the bonding area on the tooth surface was divided into 96 smaller areas. Each small area represented one unit with a surface area of 0.126 mm^2^. The total bonded surface area was 12.096 mm^2^, which was equal to the base area of the bracket. The surface area covered by adhesive remnants was calculated in mm^2^ and was reported in percentage.

The ARI score, described by Artun and Bergland [18], was calculated, as score 0 indicated no adhesive remnant on the enamel surface, score 1 indicated that less than half of the adhesive was remaining on the surface, score 2 indicated that more than half of the adhesive was remaining on the surface, and score 3 indicated that the entire adhesive was left on the surface. The composite and adhesive remnants were removed and the enamel surfaces were polished using a low-speed handpiece (operating at 30,000 rpm) and a tungsten carbide bur (Dentaurum, Ispringen, Germany) under water coolant [19]. The teeth were observed again under the microscope and the frequency, length, and direction of enamel cracks were studied by the same two observers.

### Statistical analysis:

The data were analyzed using SPSS version 20 software program (SPSS Inc., Chicago, IL, USA). The analysis of covariance was applied to compare the frequency and length of enamel cracks between the two groups after debonding by considering the baseline values as the covariate. Mann-U-Whitney test was applied to evaluate the differences in the ARI scores between the two groups. P<0.05 was considered statistically significant.

## RESULTS

The means and standard deviations (SD) of the frequency and length of cracks before and after acid-etching and laser-etching are presented in Table 1. The mean±SD number of cracks in the acid-etched and laser-etched groups equaled to 2.07±0.333 and 1.93±0.509, respectively. The mean±SD crack length in the acid-etched and laser-etched groups equaled to 12734.41±4104.42 and 11557.1±5586.056 μm, respectively. No significant difference was noted in the frequency or length of enamel cracks between the two groups before debonding (P>0.05); therefore, the two groups were identical with regards to these characteristics before the intervention. The results of the analysis of covariance showed that there were no significant differences in the length and number of cracks between the groups after the intervention (P=0.356 and 0.199, respectively). The ARI scores are presented in Table 2. The ARI scores of the acid-etched group were significantly higher than those of the laser-etched group (P<0.001).

**Table 1.** The means and standard deviations (SD) of the frequency and length (μm) of enamel cracks before and after acid-etching and laser-etching

	**Before acid-etching**	**After acid-etching**	**Before laser irradiation**	**After laser irradiation**
**Number of cracks**	0.20 ± 0.08	2.07 ± 0.33	0.24 ± 0.12	1.93 ± 0.51
**Length of cracks**	8096.41 ± 4552.75	12734.41 ± 4104.42	8255.61 ± 4816.56	11557.10 ± 5586.06

**Table 2.** Frequency distribution of the ARI scores in the two groups

**Groups**	**Score 0**	**Score 1**	**Score 2**	**Score 3**
Acid-etching	5	5	4	16
Laser irradiation	11	14	5	0

## DISCUSSION

Direct bracket bonding offers many benefits in contemporary orthodontics; however, the enamel surface preparation method and type of adhesive can significantly affect the bracket bonding. As explained by Martinez-Insua et al [4] in 2000, conventional acid-etching has several disadvantages including removal of the superficial protective enamel layer and demineralization, which make the teeth more vulnerable to long-standing acid attacks. This is especially important when the acid-etched surface is not entirely covered by resin and is exposed to saliva. Considering the shortcomings of acid-etching, Ozer et al in 2008 [6], and Lee et al in 2003 [8], introduced laser-etching as a suitable alternative to acid-etching of the enamel surface. In the current study, the frequency and length of enamel cracks in the buccal surface and the ARI scores were compared between the two groups of teeth subjected to acid-etching and laser-etching. The results revealed no significant difference in terms of the length or number of cracks between the two groups after orthodontic bracket debonding. The fragility of enamel depends on the age of the patient since the organic and mineral contents of the enamel surface change with aging; thus, the extracted teeth of 13–19-year-old patients were used in the current study due to low susceptibility to fracture [20]. A search of the literature yielded no previous study on the effect of laser-etching of the enamel surface prior to bracket bonding on the frequency and number of enamel cracks after debonding. Thus, we compared our findings with those of the previous studies on the bond strength following laser-etching and acid-etching. Several studies have evaluated the efficacy of enamel surface preparation with laser prior to orthodontic bracket bonding.

The morphological changes in the enamel caused by laser irradiation depend on the energy density of the laser, duration of exposure, distance of the laser handpiece from the surface and frequency of water and air spraying [21,22]. In the current study, the ARI scores were also compared between the two groups. According to Mann-U-Whitney test, a significant difference in the mean rank of the ARI score was found between the two groups and a lower value was observed in the laser-etched group. In other words, less adhesive remained on the enamel surface in this group, which is in line with the results of the study by Hosseini et al [17] in 2012, but in contrast to those of the study by Gokcelik et al [23] in 2007, since the latter showed higher ARI scores in the Er:YAG laser-etched samples compared to that in the acid-etched group. The difference between our results and those of Gokcelik et al [23] is probably due to the different types of the applied lasers. In our study, based on the ARI scores, debonding mainly occurred at the resin-enamel interface, leaving less adhesive remnant on the enamel surface in the laser-etched group; therefore, less time is needed for resin removal with a lower risk of damaging the enamel surface. Thus, this type of bonding is clinically favorable [1]. It should be noted that debonding at the resin-enamel interface has a higher frequency in the clinical setting compared to the in-vitro conditions because the factors in the oral environment such as thermal changes, humidity, temperature and microbial plaque compromise the enamel-etching and decrease its efficacy [24]. Moreover, the structural pattern of the bracket base is designed in such a way that debonding is uncommon at the resin-bracket interface [25]. In contrast to the current results, Lee et al [8] observed that the teeth prepared with acid-etching or Er:YAG laser irradiation showed a higher frequency of adhesive fractures at the resin-bracket interface. Such a difference in the results may be attributed to the different types of tests since Lee et al [8] performed tensile bond strength test. Similarly, Valletta et al [26] reported that debonding occurred mainly at the bracket-resin interface during tensile bond strength testing and at the resin-tooth interface in shear bond strength testing, which were in line with our findings. In contrast to our results, Fernandez and Canut [24] observed a higher frequency of bond failure at the bracket-resin interface. Proffit et al [27] stated that the greatest damage to the enamel occurs after debonding at the enamel-resin interface, which is in contrast to our findings. In previous studies [15,28], in order to observe enamel cracks and measure their lengths, the teeth had been fixed in only one direction and illuminated from another direction under a microscope; thus, only the cracks perpendicular to the direction of the light rays were visualized, while in the present study, the teeth were rotated 360° around the center of their buccal surfaces to detect all enamel cracks with different orientations. In this method, the whole length of enamel cracks, even curved cracks, was recorded. Also, we had a relatively large sample size, which increased the reliability of our findings. These were among the strong points of the current study. However, the current study had an in-vitro design. In-vitro studies cannot completely simulate the oral clinical environment in terms of thermal changes, humidity, acid attacks and microbial plaque. Moreover, the force applied to the brackets under the laboratory conditions is different from that in the clinical setting.

Thus, the generalization of in-vitro results to the clinical setting must be done with caution. Adhesive failure at the enamel-adhesive interface, although favorable in terms of leaving minimal adhesive remnants on the enamel, may negatively affect the shear bond strength in the laser-etched samples. Thus, this issue must be investigated in future studies. Also, further studies are recommended to find the most suitable settings of Er,Cr:YSGG laser irradiation to obtain the most favorable results.

## CONCLUSION

Within the limitations of this study, no significant difference was noted in the frequency or length of enamel cracks after bracket debonding between the two groups of laser-etching and acid-etching. Therefore, by considering the side effects of acid-etching (demineralization and formation of white spot lesions), Er,Cr:YSGG laser irradiation with the exposure settings applied in this study is recommended as an efficient alternative to acid-etching for enamel surface preparation prior to bracket bonding.
